# Access to Essential Medicines in Low- and Middle-Income Countries: A Systematic Review of Barriers and Facilitators

**DOI:** 10.3389/ijph.2026.1608754

**Published:** 2026-01-23

**Authors:** Israel Herrera-Ramirez, Emanuel Orozco-Nuñez, Germán Guerra, Anahi Dreser-Mansilla, Raul Enrique Molina-Salazar

**Affiliations:** 1 Center for Health Systems Research, National Institute of Public Health, Cuernavaca, Mexico; 2 Department of Economics, Universidad Autónoma Metropolitana Iztapalapa, Mexico City, Mexico

**Keywords:** access to medicines, affordability, appropriate use, availability, health governance, essential medicines, low- and middle-income countries

## Abstract

**Objectives:**

To conduct a systematic review to analyze the barriers and facilitators related to accessing essential medicines in low- and middle-income countries (LMICs).

**Methods:**

We searched PubMed, SciELO, LILACS, and Web of Science for studies published between 2002 and 2025. Studies were included if they were peer-reviewed, written in English or Spanish, and reported data on barriers or facilitators across three dimensions: availability, affordability, and adequate use.

**Results:**

From 1010 identified records, 36 studies were included. Most were quantitative (n = 26), followed by qualitative (n = 8) and mixed-methods (n = 2) designs. Barriers (n = 34 studies) were reported more frequently than facilitators (n = 25), particularly for availability and affordability. Key barriers included public sector stock-outs and high prices in the private sector. A key facilitator was the presence of a national essential medicines list.

**Conclusion:**

Our analysis compiles evidence on barriers and facilitators affecting access to essential medicines in LMICs. Policies favoring generic drug procurement and public–private sector disparities highlight the complexity of ensuring equitable access.

## Introduction

The importance attributed to access to medicines and their impact on health through adequate medical treatments is a key point of interest within the field of health systems research [1]. Specifically, the conversation revolves around access to medicines that meet the priority health needs of a population, commonly known as essential medicines [2, 3]. According to Bigdeli et al., access to essential medicines considers three fundamental dimensions: availability, affordability, and adequate use of medicines [3]. Under this framework, the understanding of access to essential medicines requires the analysis of these three dimensions, as well as their relationship with the other constituent elements that make up the health system [3, 4].

The term ‘access’ is multifaceted and encompasses three dimensions. Firstly, availability of essential medicines involves their continuous presence and accessibility within functional healthcare systems, closely tied to their selection based on the healthcare needs of the majority of the population, as well as the quality, safety, and cost-effectiveness of selected medicines [3, 5]. Ensuring availability depends on factors like healthcare capacity, infrastructure, and efficient storage and distribution to both private and public dispensing centers [5]. Secondly, affordability is the ability to obtain medicines without financial burden and can be broadly assessed at two levels: the patient/household level and the health system level [3]. Factors can, therefore, range from high household spending on medicines, unfavorable financial coping strategies, and disproportionate allocation of household resources, to aspects such as medicine prices, budget impact, and equitable resource distribution to meet the needs of all population groups. Finally, appropriate use has been defined as the situation when “patients receive medications appropriate to their clinical needs, in doses that meet their own individual requirements, for an adequate period of time, and at the lowest cost to them and their community” [2]. As such, understanding each dimension mentioned is relevant, but recognizing their interconnectedness is equally important. This involves recognizing how each dimension affects and is affected by others, as well as its influence on the broader building blocks of the health system.

Access to essential medicines is a growing global concern that varies by context, affecting both low- and middle-income countries (LMICs) and high-income countries [6]. These challenges may stem from system-wide differences, including limited financial support, inadequate allocation and use of resources, infrastructure and human resource constraints, weak governance and policy frameworks [6], and budget shortages [7]. A multi-country study found that public-sector availability of generic medicines ranged from 29.4% to 54.4% across WHO regions, consistently lower than in the private sector [8]. The same study showed that 1 month of treatment for three common chronic noncommunicable diseases was unaffordable for a large share of the population when purchased privately [8]. Similarly, a study of private pharmacy prices for four commonly used cardiovascular medicines across 18 countries reported potential unaffordability, varying by country [9].

The situation regarding appropriate medicine use in LMICs remains unclear and largely speculative due to limited research and the involvement of multiple actors [10]. Nevertheless, a study in Brazil identified challenges in appropriate medicine use, reporting that nearly half of respondents engaged in at least one form of inappropriate use, including non-adherence, obtaining prescriptions from unauthorized sources, and improper storage [11]. Although much of the literature focuses on developing countries, access barriers have also been documented in developed countries, particularly related to access to specialists and socioeconomic differences in prescribing [12]. However, gaps between LMICs and developed countries persist, with availability of antihypertensive medicines reported at 13% versus 94% in healthcare facilities [13].

The interconnectivity among availability, affordability and appropriate use can give rise to various barriers and facilitators to accessing essential medicines, highlighting the need for its analysis to establish a robust governance framework. As a function, governance ensures strategic policy frameworks, effective oversight, coalition-building, regulation, system design, and accountability [14]. Within a pharmaceutical system, it functions as a central hub, interacting with all its components, aimed at ensuring access to medicines within health systems [4]. Weak governance adversely affects this system, as evidenced by its potential to facilitate corruption or participate in unethical practices [15, 16]. Consequently, understanding of governance elements is necessary in order to assess their impact on pharmaceutical system performance, which can be achieved by identifying barriers and facilitators that influence access to essential medicines.

Given governance’s role in ensuring access to essential medicines, particularly in LMICs facing distinct challenges, conducting a comprehensive systematic review is essential for analyzing mechanisms in accessing these medicines [8, 17–19]. A comprehensive analysis is needed, which requires systematically identifying and describing barriers and facilitators across all dimensions, while highlighting their interconnections within health and pharmaceutical systems. Our objective is to bridge this gap by integrating current peer-reviewed literature through a systemized literature review [20], aiming to enhance our understanding of factors that facilitate or impede access to essential medicines in LMICs.

## Methods

We followed the PRISMA guidelines [21] and electronically searched PubMed, SciELO, LILACS, and Web of Science databases between 1 January 2002 and 7 Nov 2025 Although we tailored the specific search strategy to the requirements of each database, we used three main keywords to guide the general database search: LMIC, access, and essential medicines. We decided to use the MeSH term “Developing Countries” for LMIC, which effectively includes all LMIC while leaving out HICs in the search. For the keyword “access”, we used the MeSH term “Health Services Accessibility”, which refers to the ability to gain entry to and receive care and services from the healthcare system. This also included “access to medicines” and other variations as its entry terms. Finally, for the keyword “essential medicines,” we used the designated MeSh term “Essential Drugs,” which is defined as drugs considered essential to meet the health needs of a population as well as to control drug costs. We also used their equivalent *Health Science Descriptors* or DeCS (for its acronym in Spanish) when conducting the search in Spanish. The detailed and verbatim search strategies used for each database are presented in Supplementary Material 1.

### Defining Barriers and Facilitators in Access to Essential Medicines

Systematic reviews that document barriers and facilitators to various outcomes are essential for understanding factors that influence transformation and change processes. A key recommendation from Bach-Mortensen and Verboom is to explicitly define the studied factors (barriers and facilitators) and ideally, clarify the presumed characteristics of these terms. While achieving clarity is inherently difficult, especially within the complexities of medicine access, we`ve established the following general definition for the purpose of this article: barriers are hindrances or challenges impeding desired outcomes in access to essential medicines, while facilitators are identified as factors or resources easing the achievement of desired outcomes. These definitions apply to any aspect of availability, affordability, and appropriate use of essential medicines [3].

Following this definition and Bigdeli’s conceptualization of access to essential medicines [3], our data extraction categorized information into barriers and facilitators, each subdivided into three categories representing availability, affordability, and appropriate medicine use. Recognizing medicines as one of the building blocks of health systems [22], we can align the overarching goals of these previously mentioned dimensions with established health system objectives, which encompass health improvement, patient-centeredness, and financial protection. Therefore, barriers and facilitators in this study can also include factors that influence pharmaceutical system performance [4], which subsequently impact intermediate and final health system objectives.

### Eligibility Criteria

During screening and eligibility stages, we applied the following inclusion criteria:

- Studies published after 1 January 2002;
- Studies written in English or Spanish;
- Original research peer-reviewed articles;
- Studies conducted in at least one country categorized by the World Bank Group as low income, low middle-income, or high-middle income;
- Studies that included data or information regarding barriers or facilitators in accessing essential medicines as conceptualized by Bigdeli et al. [3] and;
- Studies that were identified as qualitative, quantitative, or mixed method studies.

We excluded papers that were not original research studies, such as commentaries, editorials, correspondences, letters to the editor, essays, short articles, and preprint articles. We excluded studies focused solely on high-income countries or those where it was unclear whether they provided information on access to medicines in LMICs. Additionally, studies were excluded if essential drugs were not a primary focus or if they did not report outcomes related to access to essential medicines. Text that could not be retrieved through conventional methods was also excluded.

### Screening and Eligibility Stage

We managed all citations during each stage through the reference management software Zotero (version 6.0.20). Zotero served to identify and remove duplicates, screen based on pre-established criteria, and download/import full-text versions of identified studies. Before the initial screening, we removed records due to duplicity, language, or publication date. Initial screening then began based on Title and Abstract. We conducted further exclusion by pre-established inclusion and exclusion criteria once we retrieved full-text versions of the studies.

### Data Extraction and Analysis

We extracted the main study parameters into a summary Excel table, which included title, authors, year of publication, objective, methodology overview, study type, country of study, and a summary of findings and outcomes reported. For outcomes reported, we categorized our findings into two general categories: barriers and facilitators. In each of these categories, we further disaggregated our findings into 3 subcategories: availability, affordability, and adequate use.

## Results

**FIGURE 1:**
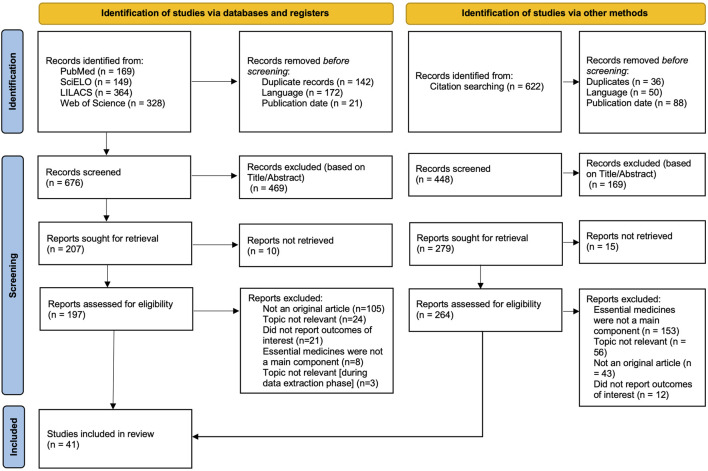
Preferred Reporting Items for Systematic Reviews and Meta-Analyses (PRISMA) flow diagram (Review, low- and middle- income countries, 2002–2025).

The study’s selection process is depicted in the PRISMA flow diagram as seen in Figure 1. 1010 records in total were identified and reduced to 36 full-text documents after screening, extraction and analyzed for this review.

With regard to location, 19 studies focused on a single country, with a notable concentration in Brazil (n = 6), while 17 studies were multi-country analyses. When categorized by their methodological approach, most were quantitative (n = 26, 72.2%), with a smaller number adopting a qualitative design (n = 8, 22.2%) and two mixed-methods studies (n = 2, 5.6%). The quantitative studies (mostly cross-sectional) primarily measure the extent of access barriers, with special emphasis to indicators of availability and affordability, usually together, while the qualitative studies explain the underlying policy, systemic, and perceptual drivers. An overview of the included studies is provided in Table 1.

**TABLE 1:** Overview of key study characteristics included in the review (Review, low- and middle- income countries, 2002–2025).

**Title**	**Author**	**Year**	**Objective**	**Methodology Overview**	**Countries**
**Quantitative approach**					
Public and private sector responses to essential drugs policies: a multilevel analysis of drug prescription and selling practices in Mali	Maiga et al.	2003	Compare prescribing and selling practices in Mali based on public sector contributions to drug supply.	Quantitative study using multilevel models to analyze content and cost of medication transactions in Mali.	Mali
The availability and affordability of selected essential medicines for chronic diseases in six low- and middle-income countries.	Mendis et al.	2007	Assess availability and affordability of medicines for chronic diseases in six low- and middle-income countries.	Quantitative survey analyzing availability, price, and affordability of 32 medicines in Bangladesh, Brazil, Malawi, Nepal, Pakistan, and Sri Lanka.	Bangladesh, Brazil, Malawi, Nepal, Pakistan and Sri Lanka
Medicine prices, availability, and affordability in 36 developing and middle-income countries: a secondary analysis.	Cameron et al.	2009	Present a secondary analysis of medicine availability, price, and affordability in 45 national and subnational surveys.	Quantitative study with WHO/HAI methodology, adjusting data from 45 surveys in 36 countries.	Armenia, Brazil, Cameroon, Chad, China, El Salvador, Ethiopia, Fiji, Ghana, India, Indonesia, Jordan, Kazakhstan, Kenya, Kuwait, Kyrgyzstan, Lebanon, Malaysia, Mali, Mongolia, Morocco, Nigeria, Pakistan, Peru, Philippines, South Africa, Sri Lanka, Sudan, Syria, Tajikistan, Tanzania, Tunisia, Uganda, United Arab Emirates, Uzbekistan, and Yemen.
Measuring medicine prices in Peru: validation of key aspects of WHO/HAI survey methodology	Madden et al.	2010	Evaluate the potential for bias arising from the limited list of medicines and the geographical sampling used in the WHO/HAI survey methodology, and validate key aspects by comparing retail prices with independent wholesale prices.	Quantitative study using an expanded sample of pharmaceutical outlets (including remote areas) based on the WHO/HAI methodology, comparing the data with international reference prices and IMS Health data.	Peru
Availability, price and affordability of cardiovascular medicines: A comparison across 36 countries using WHO/HAI data	van Mourik et al.	2010	To examine the availability, pricing, and affordability of chronic-care cardiovascular medicines in developing countries using standardized WHO/HAI data.	Secondary quantitative analysis of WHO/HAI survey data measuring percentage availability, median price ratios (adjusted for inflation and purchasing power), and affordability in days' wages.	Armenia, Brazil, Cameroon, Chad, China, El Salvador, Ethiopia, Fiji, Ghana, India, Indonesia, Jordan, Kazakhstan, Kenya, Kuwait, Kyrgyzstan, Lebanon, Malaysia, Mali, Mongolia, Morocco, Nigeria, Pakistan, Peru, Philippines, South Africa, Sri Lanka, Sudan, Syria, Tajikistan, Tanzania, Tunisia, Uganda, United Arab Emirates, Uzbekistan, and Yemen.
Is the Brazilian pharmaceutical policy ensuring population access to essential medicines?	Bertoldi et al.	2012	To evaluate medicine prices, availability, and affordability in the Brazilian state of Rio Grande do Sul, comparing originator brands, generics, and similar medicines across public, private, and “popular” pharmacies.	Quantitative survey using the WHO/HAI methodology. Data on prices and availability of 50 medicines were collected in 56 pharmacy outlets (public, private, and “popular” pharmacies) across six cities in Southern Brazil.	Brazil
Availability, prices and affordability of essential medicines in Haiti	Chahal et al.	2013	Determine availability, prices, and affordability of essential medicines in Haiti.	Cross-sectional nationwide survey in 2011, using WHO/HAI methodology in 163 medicine outlets.	Haiti
WHO essential medicines policies and use in developing and transitional countries: an analysis of reported policy implementation and medicines use surveys.	Holloway and Henry	2014	Determine the association between WHO essential medicines policies and quality use of medicines.	Quantitative study correlating reported implementation of WHO essential medicines policies with quality use of medicines.	Angola, Armenia, Bahrain, Bolivia, Brazil, Burkina Faso, Burundi, Cambodia, Cameroon, Chile, China, Colombia, Congo, Cuba, Democratic Republic of the Congo, Egypt, Ethiopia, Gambia, Ghana, Guatemala, Guinea, India, Indonesia, Iran, Jordan, Kenya, Kyrgyzstan, Lao People's Democratic Republic, Malawi, Malaysia, Mali, Mongolia, Morocco, Mozambique, Namibia, Nepal, Niger, Nigeria, Oman, Pakistan, Peru, Philippines, Rwanda, Samoa, Senegal, Serbia and Montenegro, South Africa, Sudan, Tanzania, Thailand, Tonga, Tunisia, Uganda, Uzbekistan, Vietnam and Zambia
Evaluating medicines prices, availability, affordability and price components in Sudan	Kheder and Ali	2014	To measure medicines prices, availability, affordability, and price components in different sectors in Sudan to assess the impact of pricing policies.	A quantitative field study using the standardized WHO/HAI methodology, surveying 50 medicines in public and private outlets across six geographical regions from March 2012 to April 2013.	Sudan
The Effects of Intellectual Property Rights on Access to Medicines and Catastrophic Expenditure	Jung and Kwon.	2015	Investigate the effect of intellectual property rights on access to medicines and catastrophic expenditure.	Quantitative study using World Health Surveys 2002–2003 data, measuring IPR protection level and adjusting for individual and country characteristics.	Bangladesh, Brazil, Burkina Faso, Chad, China, Congo, Dominican Republic, Ecuador, Ethiopia, Ghana, Guatemala, India, Kenya, Malawi, Malaysia, Mali, Mauritania, Mauritius, Mexico, Morocco, Nepal, Pakistan, Paraguay, Philippines, Russia, Senegal, South Africa, Sri Lanka, Swaziland, Tunisia, Ukraine, Uruguay, Vietnam, Zambia and Zimbabwe
Access to medicines for chronic diseases in Brazil: a multidimensional approach	Auxiliadora-Oliveira et al.	2016	Analyze access to medicines for non-communicable diseases in Brazil based on socioeconomic factors.	Analysis of PNAUM data with focus on dimensions: availability, geographic accessibility, acceptability, affordability.	Brazil
Progress in increasing affordability of medicines for non-communicable diseases since the introduction of mandatory health insurance in the Republic of Moldova.	Ferrario et al.	2016	Assess progress in improving affordability of medicines in Moldova since the introduction of mandatory health insurance.	Quantitative analysis using national health insurance data to estimate affordability of partially reimbursed medicines.	Republic of Moldova
Pain Treatment Continues To Be Inaccessible for Many Patients Around the Globe: Second Phase of Opioid Price Watch, a Cross-Sectional Study To Monitor the Prices of Opioids.	Pastrana et al.	2016	Second phase of a global project monitoring dispensing price of opioids for availability and affordability analysis.	Quantitative analysis of opioid dispensing prices in multiple income categories from licensed pharmacies.	Albania, Armenia, Bahamas, Bangladesh, Benin, Brazil, Canada, Colombia, Costa Rica, Dominican Republic, Egypt, El Salvador, Guatemala, Hungary, India, Iran, Ireland, Kazakhstan, Kenya, Lebanon, Lithuania, Mauritania, Mexico, Moldova, Nepal, Netherlands, New Zealand, Nigeria, Norway, Panama, Poland, Portugal, Rwanda, Samoa, Saudi Arabia, Sweden, Thailand, Togo, Uganda, United Kingdom, United States, Vietnam, and Zambia.
Free access to medicines for the treatment of chronic diseases in Brazil	Tavares et al.	2016	Analyze free access to medicines for chronic diseases in the Brazilian population.	Quantitative analysis of PNAUM data, examining prevalence of free access to medicines based on demographic and socioeconomic factors.	Brazil
Access to medicines by patients of the primary health care in the Brazilian Unified Health System	Álvares et al.	2017	Evaluate access to medicines in primary health care of the Brazilian Unified Health System from patients' perspective.	Cross-sectional study using PNAUM data with evaluations based on Penshansky and Thomas dimensions.	Brazil
Characterization of the selection of medicines for the Brazilian primary health care	Karnikowski et al.	2017	Characterize the selection process of medicines for primary health care in Brazilian regions.	Part of PNAUM, cross-sectional study with interviews of pharmaceutical service stakeholders.	Brazil
Access to medicines and diagnostic tests integral in the management of diabetes mellitus and cardiovascular diseases in Uganda: insights from the ACCODAD study.	Kibirige et al.	2017	Assess availability, cost, and affordability of diabetes and asthma medicines in Uganda.	Quantitative study in 22 public hospitals, 23 private hospitals, and 100 private pharmacies.	Uganda
Availability of essential medicines in primary health care of the Brazilian Unified Health System	Rezende Macedo do Nascimento et al.	2017	Characterize the availability of tracer medicines in primary health care of the Brazilian Unified Health System.	Quantitative analysis of medicine availability in primary health care using Rename items and observation scripts.	Brazil
Evaluating access to essential medicines for treating childhood cancers: a medicines availability, price and affordability study in New Delhi, India	Faruqui et al.	2019	Assess the availability, price and affordability of essential medicines for treating childhood cancers in New Delhi, India.	Cross-sectional survey using modified WHO/HAI methodology in 7 survey anchor hospitals (public and private) and 32 retail pharmacies to analyze availability and median price ratios of 33 anti-neoplastic medicines.	India
Access to Cardiovascular Disease and Hypertension Medicines in Developing Countries: An Analysis of Essential Medicine Lists, Price, Availability, and Affordability.	Husain et al.	2020	Analyze country EMLs for CVD and hypertension medicines, assess availability, price, and affordability globally.	WHO online repository and surveys in 59 countries for quantitative analysis.	53 countries were included in the analysis of national Essential Medicines Lists (NEML) and 59 countries (from 84 surveys) were included in the analysis of medicine price, availability, and affordabilit.
Acceso a medicamentos en pacientes del Seguro Integral de Salud (SIS) con diabetes mellitus y/o hipertensión arterial en Perú	Espinoza-Marchan et al.	2021	Analyze access to medicines in SIS-affiliated patients with diabetes and/or hypertension in Peru.	Cross-sectional descriptive study using WHO-adapted surveys in Cajamarca, Trujillo, and Callao.	Peru
Access to cancer medicines deemed essential by oncologists in 82 countries: an international, cross-sectional survey	Fundytus et al.	2021	Investigate alignment of cancer medicines in EML with oncologists' priorities globally and assess accessibility.	International cross-sectional survey with global oncologists, exploring availability and cost.	The study included 82 countries. The study analyzes them by stratifying them into the following: Low-income and lower-middle-income countries, upper-middle-income countries y high-income countries.
Pharmaceutical procurement among public sector procurers in CARICOM	Preston et al.	2021	Examine medicines in CARICOM procurement markets, including manufacturer details and affordability factors.	Quantitative analysis of procurement information from CARICOM procurers.	The study was conducted using data from selected public sector procurers in the Caribbean Community (CARICOM), which consists of 20 English-speaking governments, plus Haiti and Suriname.. The article anonymized the four specific procurers (A, B, C, and D).
Availability and accessibility of opioids for pain and palliative care in Colombia: a survey study	Ximena-León et al.	2021	Identify barriers to opioid availability and accessibility for pain and palliative care in Colombia.	A cross-sectional study using an online survey distributed to 1,208 Colombian prescribers. The analysis used descriptive statistics (relative frequencies) and Fisher's exact test to measure significance.	Colombia
Public Programs for Essential Medicine Access in a Small Municipality: A Cross-Sectional Analysis	Chaves et al.	2022	To describe the sociodemographic profile and the medication and health service usage of patients with systemic arterial hypertension and/or diabetes mellitus in a small municipality who use the public medication access programs Health has no Price (Saúde Não Tem Preço - SNTP) and the Minas Pharmacy Network.	A study conducted in 2019 with 341 participants. Home interviews were conducted with patients with hypertension and/or diabetes using a standardized, semi-structured questionnaire.	Brazil
Access to and Affordability of World Health Organization Essential Medicines for Cancer in Sub-Saharan Africa: Examples from Kenya, Rwanda, and Uganda	Kizub et al.	2022	Evaluate cancer medicine access in Sub-Saharan Africa based on essential medicine lists and affordability.	Population, healthcare financing, minimum wage, cancer data used for quantitative analysis across multiple countries.	Kenya, Rwanda, and Uganda
Public policy coverage and access to medicines in Brazil	Moraes et al.	2022	To describe consumption patterns for monetary and non-monetary acquisition of medicines according to age and income groups, highlighting pharmaceuticals associated with health programs with specific access guarantees	A descriptive observational study using microdata from the 2017-2018 Pesquisa de Orçamentos Familiares (Household Budget Survey, POF/IBGE). The study reviewed Brazilian health programs with specific medicine access guarantees, matched them to pharmaceutical products listed in the POF questionnaire, and then described the frequencies and percentages of monetary vs. non-monetary acquisition by age and income groups	Brazil
Outpatient pharmaceutical office: access to medicines in public health	Morgado Junior et al.	2023	To evaluate the implementation of an outpatient pharmaceutical office in a teaching hospital regarding access to medicines available in the Brazilian Unified Health System (SUS).	A descriptive-analytical study based on the secondary data analysis of 735 pharmaceutical appointments conducted from 2015 to 2017.	Brazil
Health technology assessment in the Brazilian National Health System: profile of CONITEC exclusion recommendations, 2012-2023	Pinheiro et al.	2024	To analyze the recommendations for exclusion of health technologies in the Brazilian National Health System (SUS) made by CONITEC from 2012 to 2023, and to identify the disinvestment criteria used.	A documentary, descriptive, and retrospective analysis of CONITEC recommendation reports that assessed requests for technology exclusion.	Brazil
Time to inclusion of selected medicines for priority diseases in National Essential Medicines Lists compared with the WHO Model List.	Hellamand et al.	2025	To assess the time it took for countries to adjust their National Essential Medicines Lists (NEMLs) when medicines were added or deleted from the WHO Model List, and to determine if this differed between selected priority diseases.	A descriptive study that extracted medicines added or deleted from the WHO Model List between 2007 and 2021 for five priority diseases (diabetes, hepatitis C, HIV, oncology, and tuberculosis). This list was then compared against the NEMLs or reimbursement lists (RLs) from 20 purposefully selected countries. The analysis assessed the time to inclusion in the national lists and the percentage of medicines included in the most recent list.	The study included 20 countries. Low-income: Ethiopia, Rwanda, Uganda, Zambia Lower middle-income: Bhutan, India, Lebanon, Nigeria, Pakistan, The Philippines Upper middle-income: Brazil, Jordan, Malaysia, The Russian Federation, South Africa, Suriname High-income: Australia, Denmark, Ireland, Uruguay
Prices and Affordability of Essential Medicines in 72 Low-, Middle-, and High-Income Markets.	Wouters et al.	2025	To compare the list prices and affordability of essential medicines across high-, middle-, and low-income markets.	A cross-sectional study using 2022 data from IQVIA on the list prices and volumes of 549 essential medicines in 72 markets. It used Laspeyres price indices to compare prices and calculated the number of days' minimum wage needed to pay for one month of treatment for 8 specific medicines to assess affordability.	The study analyzed data from 72 high-, middle-, and low-income markets, covering a total of 87 countries. 40 high-income markets (39 countries plus Hong Kong) 32 middle-income markets (42 countries) 1 low-income market (6 countries)
	**Qualitative approach**				
Drug supply strategies, constraints and prospects in Nigeria.	Yusuff and Tayo	2004	Identify strategies for public drug supply in Nigeria and assess functionality.	Qualitative study with semi-structured interviews at Department of Food & Drugs, Drug procurement unit in Nigeria.	Nigeria
Access to Essential Medicines in Pakistan: Policy and Health Systems Research Concerns	Zaidi et al.	2013	Identify policy concerns in access to medicines in Pakistan and present prioritized concerns.	Exploratory research using WHO Framework, key informant interviews, literature review.	Pakistan
Strategic contracting practices to improve procurement of health commodities.	Arney et al.	2014	Offer an overview of VA and DOD procurement practices and recommend strategic procurement practices for developing countries.	Qualitative study involving literature reviews, interviews, and evaluation of procurement practices' suitability for developing countries.	Ghana, Kenya, Mozambique, Rwanda, Tanzania, Uganda and Zambia
Acceso a medicamentos de alto precio en Brasil: la perspectiva de médicos, farmacéuticos y usuarios	Mattozo-Rover et al.	2016	Explore perceptions on access to medication supplied by CEAF in the Brazilian Unified Health System.	Descriptive, qualitative study with focal group and interviews in Santa Catarina.	Brazil
Legislating for universal access to medicines: a rights-based cross-national comparison of UHC laws in 16 countries.	Perehudoff et al.	2019	Develop and apply an assessment tool to UHC legislation in 16 mostly LMICs for identifying legal texts promoting access to medicines.	Qualitative cross-national study analyzing UHC legislation in 16 countries against an assessment tool with 12 principles.	Algeria, Chile, Colombia, Ghana, Indonesia, Jordan, Mexico, Morocco, Nigeria, Philippines, Rwanda, South Africa, Tanzania, Tunisia, Turkey and Uruguay
How does performance-based financing affect the availability of essential medicines in Cameroon? A qualitative study.	Sieleunou et al.	2019	Explore how Performance-based financing in Cameroon influences essential medicines availability.	Qualitative study with in-depth interviews of health services managers, healthcare providers, and community members in Cameroon.	Cameroon
Access to Controlled Medicines in Low-Income Countries: Listening to Stakeholders in the Field.	Vitry et al.	2021	Examine practices and challenges in the legal trade of controlled medicines in 3 African countries.	Qualitative survey with semi-structured interviews of stakeholders engaged in the trade.	Democratic Republic of the Congo (DRC), Kenya and Uganda
The end of patent extensions and the Productive Development Partnerships: effects on access to medicines in Brazil	Lopes et al.	2024	To analyze the effect of the Brazilian Supreme Court's judicial decision in ADI 5529/DF (which ended automatic patent extensions) on patent applications and patents for 15 drugs relevant to the Productive Development Partnerships (PDPs).	A documentary case study analyzing the status of 90 patent applications related to 15 PDP drugs as of December 31, 2020. Data was collected from the websites of the National Institute of Industrial Property (INPI), the Ministry of Health, ANVISA, and the Brazilian Medicines Market Regulation Chamber (CMED).	Brazil
	**Mixed method approach**				
La política farmacêutica nacional en Colombia y la reforma de la seguridad social: acceso y uso racional de medicamentos	Restrepo et al.	2002	Analyze whether pharmaceutical policy formulation promotes accessibility, availability, and rational use of medicines in Colombia.	Mixed methods approach involving macro and micro perspectives, studying legal framework and drug supply system.	Colombia
Importance of medicine quality in achieving universal health coverage	Ozawa et al.	2020	Assess the importance of ensuring medicine quality for universal health coverage.	Mixed-method study developing a systems map connecting medicines quality assurance with UHC goals.	A quantitative regression analysis using data from 63 low- and middle-income countries (LMICs).and a focused health and economic modeling case study on four sub-Saharan African countries: the Democratic Republic of the Congo (DRC), Nigeria, Uganda, and Zambia.

The studies presented in Table 1 show that the most frequently discussed topics were improving access through price controls (e.g., securing purchase prices that do not restrict access to medicines), followed by processes and policies aimed at expanding access to medicines. To a lesser extent, medicine lists based on health priorities and price improvements to increase purchasing power were highlighted. These first three topics accounted for more than 60% of the selected articles.

Methodologically, most studies employed quantitative approaches, while political analyses and qualitative studies were less common. More than half focused on Latin America, with Brazil standing out with 12 included studies. Multicenter studies were the second most frequent category (n = 16), followed by four studies from Africa, two from Asia, and one from Europe.

**FIGURE 2:**
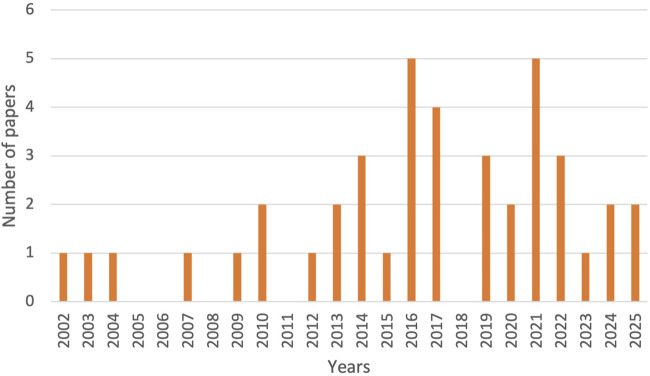
Annual distribution of published papers included in the review (Low- and Middle-Income Countries, 2002–2025).

Temporal analysis of the 36 studies (Figure 2) shows an increase in publications, with 72.2% published between 2016 and 2025. Peaks in 2016 and 2021 align with the SDGs and the COVID-19 pandemic. Disciplinary focus was dominated by Public/Global Health and Health Policy journals, with limited representation from Pharmaceutical Practice.

Regarding disciplinary focus (Table 2), the literature is dominated by broader systemic perspectives, primarily appearing in Public/Global Health (n = 16, 44.4%) and Health Policy and Systems (n = 6, 16.7%) journals, alongside a strong clinical viewpoint from General Medicine/Clinical Specialty (n = 9, 25.0%). The low representation in fields like Pharmaceutical Practice (n = 2, 5.6%) may indicate a potential gap in implementation-focused research.

**TABLE 2:** Number of papers in the sample by type of journal subject field (Low- and Middle-Income Countries, 2002–2025).

Type of journal (subject field)	Number of papers
Public/Global Health	19 (46.3%)
General Medicine/Clinical Specialty	10 (24.4%)
Health Policy and Systems	7 (17.1%)
Interdisciplinary Science	3 (7.3%)
Pharmaceutical Practice	2 (4.9%)
Total	41 (100%)

Source: Own elaboration.

Numbers in parentheses represent the percentage of papers in the sample.

**FIGURE 3:**
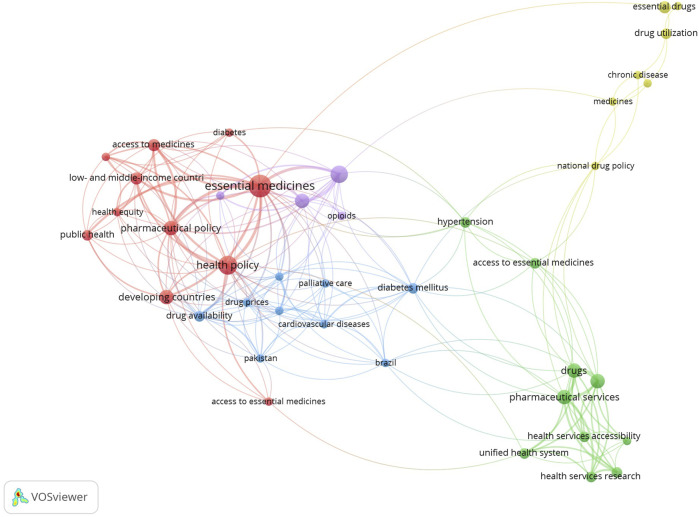
Co-occurrence network map of keywords from the included studies (Low- and Middle-Income Countries, 2002–2025).

A co-occurrence network analysis of keywords from the included studies using VOSviewer was included, which revealed four distinct thematic clusters (Figure 3). The largest cluster forms the conceptual core, linking essential medicines to health policy, access, and health equity in LMICs. The second cluster focuses on affordability, connecting drug prices with high-cost NCDs (e.g., diabetes, cardiovascular diseases) and palliative care. The third cluster addresses health systems implementation, linking pharmaceutical services to health services accessibility. Finally, the fourth cluster represents more of the evaluation aspects to essential medicines, connecting national drug policy with drug utilization for chronic diseases. These clusters map a logical flow, from the problem (equity) and barrier (e.g., price) to the mechanism or systems and outcome (utilization), in line with the thematic framework of availability, affordability, and adequate use of essential medicines.

### Barriers and Facilitators in Accessing Essential Medicines in LMIC

The majority of the studies included in this review reported findings related to barriers in different subcategories (n = 34), which is significantly higher than the number of studies that reported facilitators in any of the subcategories (n = 25). Barriers in the availability (n = 23) and affordability (n = 29) of essential medicines were the most frequently reported. Fewer studies reported on the barriers related to the adequate use of medicines (n = 13). A similar trend was observed in the facilitators category, where most studies focused on the availability (n = 17) and affordability (n = 14) dimensions, but fewer studies addressed the adequate use of medicines (n = 7). A summary of key factors is depicted in Table 3.

**TABLE 3:** Factors influencing availability, affordability and adequate use of essential medicines (Review, low- and middle- income countries, 2002–2025).

Barriers	Facilitators
Factors influencing availability	
Medicines in the private sector and brand name drugs [7, 16, 21–25]	Higher public sector availability and use of generic medicines in both public and private sectors [7, 16, 23, 26, 27] Recognition of the right to health as a state duty [28].
Selection and procurement [29, 30] • Exclusion of essential medicines from NEML • Misalignment of NEML to WHO-EML • Technical capability issues, import authorizations, and regulatory burdens • Lack of awareness among prescribers regarding procurement and therapeutic committees	Selection and procurement [29, 31, 32] • Inclusion of medicines on NEML, especially generic medicines • Alignment of NEML to WHO-EML • Fostering trustful relationships among key actors • Implementing emergency procurement procedures during crises • Establishing technical committees for essential medicines lists
General management and supply chain [32–37] • Drug stock-outs in the public sector driven by market shortages • Infrastructure, structural, and management deficiencies • Duplicity in service provision, inadequate funding, poor management, and deficient procurement systems • Deficient drug information systems • Inadequate stock maintenance • Forecast inaccuracies	General management and supply chain [22, 34, 38] • Efficient replacement of medicines or referral to alternative programs • Improved dispensing service times coupled with enhanced healthcare facility environments and cleanliness
Financing barriers [27, 39, 40] • IPR limiting access to prescribed medicines • Challenges in PBF characterized by payment delays, limited autonomy, and leadership issues • Inadequate funding • Socio-economic factors limiting adequate funding	​
Factors influencing affordability	
Purchasing of medical products in the private sector and brand-name drugs [16, 21–23, 26, 41, 42] Low household income linked to unaffordability [32, 40, 43]	Greater affordability in the public sector and in the procurement of generic medicines [26, 27, 42] Increased household income [44]
Pricing [7, 24, 25, 27, 45] • High costs of services, copayments, and fees • Variability in prices linked to diverse economic environments	Selection and procurement [29, 36, 38, 43] • Inclusion of treatments, such as cancer treatments, in the NEML and their procurement through funded UHC programs • Grant of exclusive procurement rights to the State • Establishment of a robust regulatory system streamlines procurement
Selection and procurement [29, 35] • Absence of adequate funding for treatments not included on NEML • Deficient procurement system and lack of development of a NEML leading to inefficiencies in the procurement process	Policy related factors [7, 23, 28, 32] • Development of national pharmaceutical policies governing medicines and pharmaceutical services • Policies that incentivize the use of generic medicines and fosters market competition • Transparency, participation, monitoring, accountability, pooling user contributions, international donor funding, efficient spending, financial protection for the poor, and sufficient government financing
Policy related factors [33, 36, 44] • Inadequate price control policies, unclear pricing formulas, and the proliferation of expensive originator brands • Insufficient reimbursement policies, funding constraints, and a disregard for broader health system concerns • Difficulty in securing payment authorization from health insurance companies	Clinical practice and quality of medicines [38, 46] • Good prescribing practices as a strategy in prevent polypharmacy, reducing the number of medications needed to be purchased by the patient • Investing in medicine quality as a cost-saving strategy by reducing the impact of substandard and falsified medications
Factors influencing adequate use of medicines	
Drug-Related Problems [7, 23, 30, 32, 33, 36, 42, 45] • Sharing of medications among patients with similar diagnoses • Inappropriate prescriptions • Absence of standardized protocols • Patient demands for quick cures and lenient over-the-counter access • Irrational antibiotic use • Inadequate information dissemination • Insufficient patient education	Quality in pharmaceutical care and healthcare services [22, 32, 42, 47] • Respectful and polite treatment contributes to increased patient satisfaction and adherence • Good prescribing practices the quantity of drugs dispensed • Adoption of standardized guidelines and protocols for the development of NEML • Implementation and development of national pharmaceutical policies • Health professional consultation
Quality and safety standards of medicines [43, 46] • Limited local regulatory capacity • Substandard and falsified medical products	Quality and safety standards [43] • Importation of medicines originating from more highly regulated markets ensuring higher quality and safety standards

### Barriers and Facilitators in Availability

Seven studies examined system-wide barriers affecting the availability of essential medicines, focusing on outcomes related to medicine brands and healthcare sectors (public vs. private) [8, 19, 23–26]. In Uganda, one study reported a 38.1% inadequacy in essential medicine availability, attributed to their exclusion from essential medicines lists and clinical guidelines, along with inadequate stock maintenance, inaccurate forecasting, and inefficient distribution systems [23]. Similarly, a study in Brazil identified low availability in public health units despite overall compliance rates of 70%–90%, highlighting persistent challenges in public-sector access [24]. Additional studies reinforced these findings, consistently showing lower availability in the public sector compared with the private sector, largely due to stock shortages and prescriptions for non-listed essential medicines [26, 27]. These barriers underscore systemic sector- and brand-related issues, as well as logistical challenges such as poor forecasting and distribution inefficiencies.

In contrast, five studies identified facilitating factors, including local production, the use of generic medicines, and greater availability in the private sector [8, 19, 27, 29, 31]. Four studies reported higher availability of generic medicines compared with brand-name counterparts, particularly in private facilities [8, 27, 29, 31]. One study further indicated that locally manufactured medicines had improved availability in the private sector [19]. Overall, the use of generic medicines emerges as a key facilitator, especially when supported by local production.

Several factors influencing availability are also linked to selection and procurement processes. Reported barriers include the exclusion of essential medicines from National Essential Medicines Lists (NEML) and misalignment with the World Health Organization Essential Medicines List (WHO-EML) [28, 30, 32]. Additional challenges include limited technical capacity, import authorization requirements, regulatory burdens, and lack of awareness among prescribers regarding procurement and therapeutic committees [28, 32]. Conversely, facilitators emphasize aligning NEMLs with WHO-EML recommendations, building trust among key stakeholders, and implementing emergency procurement procedures during crises [30, 32–34]. Positive contributions are also attributed to the establishment of technical committees for essential medicines lists, the inclusion of generic medicines in NEMLs, and recognition of the right to health as a state responsibility, all of which enhance selection and procurement processes [34].

General management and supply chain strategies represent another major determinant of medicine availability. Six studies identified barriers such as drug stock-outs in the public sector, often driven by market shortages and insufficient dispensing capacity [33, 35–39]. Structural and management deficiencies, including inadequate infrastructure, particularly in smaller cities, were found to affect local health service demands [35–37]. Persistent issues such as inadequate funding, weak management, inefficient procurement systems, and poor supply chain management related to low availability of centrally purchased medicines further exacerbate shortages, sometimes resulting in shifts from generic to brand-name medicines [33, 35–37]. Conversely, four studies identified facilitators within this category [24, 36, 40, 41]. Strategies such as medicine substitution and referral to alternative public programs were effective in addressing shortages [36, 40]. Improvements in dispensing service times, alongside better facility environments and cleanliness, were also associated with enhanced patient perceptions of care [24]. Together, these findings emphasize the importance of effective management and supply chain strategies, demonstrating how strengthened infrastructure, appropriate use of public programs, and system-level improvements contribute to consistent availability of essential medicines.

Financing-related barriers were also identified as impediments to essential medicine availability [31, 37, 41, 43], although none of the included studies explored facilitators within this category. Intellectual Property Rights (IPR) were highlighted as a major constraint in LMICs, restricting access to prescribed medicines and contributing to welfare losses [43]. Challenges associated with performance-based financing (PBF), including delayed payments, limited autonomy, and leadership constraints, hinder intended system improvements and contribute to inequities and fragmentation in drug management [41]. In addition, inadequate funding and poor financial management negatively affect procurement and drug information systems, leading to medicine expiration, spoilage, and stock-outs [37]. One study from Haiti presented contradictory findings, reporting low availability of the lowest-priced generic medicines [31]. However, this may be influenced by broader socioeconomic conditions, as over 75% of the population lives on less than US$2.00 per day.

### Barriers and Facilitators in Affordability

Various barriers were identified in 15 studies which suggests that the affordability of essential medicines in LMICs faces diverse challenges [8, 19, 23–27, 29, 31, 33, 42–46]. Affordability is apparently negatively affected in the private sector [8, 25, 26, 31, 44] and with brand-name drugs [19, 29, 46], posing a barrier to individuals unable to cover essential health needs. Low household affordability, as seen in the case of cancer drugs, also applies to older generic cytotoxic drugs, contributing to worsening economic strain in resource-limited settings [45]. The pricing of medicines emerges as a major factor in decreased affordability, intensified by high costs of services, copayments, and fees [19, 29, 46]. The variability in prices, which is linked to diverse economic environments, adds additional complexity to the overarching challenge of ensuring general affordability [42].

There are also reported factors that are facilitators linked to improving the general affordability landscape [29, 31, 46, 47]. Procurement of generic medicines is reported as a favorable factor, resulting in lower costs compared to brand alternatives [29]. This is particularly evident in the public sector, where generic medicines demonstrate higher affordability than their private sector counterparts according to two studies [31, 46]. As expected, increased household income has been shown to contribute to enhancing the overall affordability of essential medicines. In both the public and private sectors [47].

Under the category of selection and procurement of essential medicines, several factors affect their affordability [30, 37]. The absence of adequate funding from national governments, particularly for treatments such as cancer medicines not included in the NEML, has been associated with high out-of-pocket costs [30]. Furthermore, inadequate procurement systems combined with underdeveloped NEMLs can create inefficiencies during acquisition, consequently reducing affordability [37]. Conversely, several factors have been shown to increase affordability in four studies [30, 38, 40, 42]. The inclusion of treatments, including cancer medicines, in the NEML and their procurement through funded Universal Health Coverage (UHC) programs or government-financed mechanisms serve as important facilitators, particularly when governments hold exclusive procurement rights [30, 38, 40]. Additionally, the establishment of a regional regulatory system, such as that observed in the Caribbean region, can facilitate affordability by streamlining procurement processes and enhancing coordination through regional regulatory mechanisms [42].

In the realm of policy, multiple factors influence the affordability of essential medicines [35, 38, 47]. Key challenges include fixed price controls, unclear pricing formulas, and the widespread use of costly originator brands [35]. Barriers also stem from inadequate reimbursement policies, limited funding, and insufficient consideration of broader health system dynamics [47]. In contrast, well-developed national policies governing medicines and pharmaceutical services are consistently identified as important facilitators [27, 33]. Other facilitating factors include promoting the use of generic medicines and fostering market competition as alternatives to administrative price controls [34]. Additional facilitators include transparency and accountability mechanisms, international donor funding, financial protection for vulnerable populations, and sufficient government financing [8, 19, 34]. Overall, there is broad support for implementing pricing regulations as a key policy-guided facilitator of medicine affordability.

Finally, several factors related to clinical practice and medicine quality also affect affordability. Barriers can arise from higher disease burden and polypharmacy, where patients require multiple medicines simultaneously, increasing out-of-pocket expenditures. [40]. This is particularly evident among individuals with multiple conditions, exacerbating disparities in healthcare access. Additionally, investing in medicine quality is economically beneficial, as substandard and falsified medicines can impose substantial costs on health systems [48]. In summary, these studies highlight the importance of promoting quality, safe medicines and their appropriate use to reduce adverse effects on affordability.

### Barriers and Facilitators in the Adequate Use of Medicines

In addressing barriers related to the adequate use of medicines, findings from 10 studies spotlight several factors [8, 27, 28, 33, 35, 38, 42, 44, 46, 48]. A significant portion of these barriers pertains to drug-related problems, including the sharing of medications among patients with similar diagnoses, inappropriate prescriptions, and the absence of standardized protocols [35, 44]. These factors, combined with patient demands for quick cures and lenient over-the-counter access, collectively contribute to a compromised scenario where medicine appropriateness is jeopardized [35, 46]. Additionally, inadequate information dissemination, insufficient patient education, and non-compliance with medication sales regulations further compound the barriers to optimal medicine use [33]. Several studies also highlight the connection between appropriate medicine use and the quality and safety standards, as they are required for their effectiveness and harm reduction [42, 48].

In contrast, several facilitators were highlighted that enhance the appropriate use of medicines in five studies [24, 33, 42, 46, 49]. These factors encompass the importance of staff offering respectful and courteous care, which enhances patient satisfaction that fosters proper medicine use, effective processes for selecting essential medicines, adherence to prescription regulations, and promoting generic medication usage [24, 33, 49]. Furthermore, the combination of a prescription limiting the quantity of drugs purchased, coupled with health professional consultation, emerges as a key facilitator in mitigating risks associated with abusive consumption [46]. Regarding regulation, a study emphasized the positive impact of medicines originating from more highly regulated markets such as North American and European manufacturers. This tends to ensure higher quality and safety standards, especially for regions with limited regulatory capacity [42]. Overall, these facilitators highlight the importance of comprehensive approaches that prioritize patient education, robust regulatory frameworks, and the strengthening of regulatory capacities to ensure effective and appropriate medicine use across diverse settings.

## Discussion

To the best of our knowledge, this paper represents the first systematic review that synthesizes evidence on barriers and facilitators related to access to essential medicines in LMICs across its three interconnected dimensions: availability, affordability, and adequate use. Barriers were found to be more prevalent, particularly in the dimensions of availability and affordability, indicating a tendency to report factors that impede access rather than those that facilitate it. These findings provide evidence about the relevance of national lists and prices control and the persistence of barriers that continue impeding universal access of vulnerable populations to essential medicines.

Analysis suggests the importance of national governments developing measures for strengthening access at the local level collaborating with public and private actors. Some studies have reported improvements in availability and prices of specific medicines in various settings, including public health facilities, registered private retail medicine outlets, or through health services provided by non-governmental organizations [8, 29, 38, 44, 45]. The design of the studies varied significantly based on the particular context and available data, although the WHO/HAI methodology [50, 51] was commonly used or adapted in the included studies. Despite these efforts, in recent times about two billion people lack access to medicines, belonging to more than 80% to LMICs. Existing gaps demand innovations to improve access through economic incentives to improve availability, affordability, laws, governance, appropriate use, quality and equity [1].

It is noticeable that some studies emphasized the importance of locally manufactured medicines to improve affordability and access. To develop national production of medicines implies a coalition where governments, private sector, NGOs and robust national regulatory agencies to ensure quality and build public confidence [52, 53]. Local manufacture of medicines could require complementary components like the utilization of national surveys on medicine use, as seen in Brazil; this strategy offers extensive data on medicine access, giving testament to their value for informed decision-making [24, 28]. Analysis also suggests that these initiatives should consider sustainability mechanisms, because cases considered successful in the past like Brazil, where a high number of studies identified in this review were conducted, are facing considerable challenges of continuity attributable to governmental changes [54]. Unfortunately for the same case, overall indicators of appropriate medicine use were less commonly reported, reflecting the challenge of defining and identifying drug-related issues and the differing capacities of healthcare infrastructure and pharmacy workforce across contexts.

The outcomes related to adequate use of medicines identified in this review basically fell in one of two subcategories: assuring medicine quality and safety; and addressing drug-related problems. For instance, one of the studies reported that combating substandard medicines not only removes potentially harmful products but also saves resources [48], which enhances affordability [1]. Additionally, studies have shown the impact that drug-related problems can have on overall health, leading also to inefficiencies that increase costs for health systems [55, 56]. Low adherence is another critical aspect, as it can compromise treatment effectiveness and contribute to issues such as antimicrobial resistance [57–59].

Promoting the appropriate use of medicines is critical because it involves health providers and patients for guaranteeing that availability and affordability will generate good health outcomes if medicines are used adequately. Measuring outcomes for appropriate use poses challenges, for it requires robust procedures and a well-trained workforce to identify and prevent drug-related issues [55, 60–62]. This is because identifying and resolving DRPs involves complex tasks requiring extensive pharmacological and medical expertise. Of course, this can vary depending on the specific contexts, which presents a challenge when gathering information across different countries and making outcomes comparable [63–65]. Ultimately, ensuring the appropriate use of medicine is an essential step to truly guarantee access to medicines in any context.

A common theme observed in studies about access to medicines and this literature review was the importance of considering interconnectedness of factors influencing access to medicines, applying a systemic approach to impact various aspects of health systems. For example, the development of a NEML has consistently been identified as a facilitator in enhancing all three dimensions of access [30, 33, 40]. The selection of medicines based on the epidemiological profile of a population ensures availability by guaranteeing essential medicines can be found in health facilities, affordability as a NEML forms the basis to optimize procurement processes, resulting in lower prices, and adequacy by selecting medicines that align with clinical guidelines to promote proper medicine use [66]. Furthermore, the function of selection and procurement can fall within the functions of resource generation and financing, as mechanisms such as pooled procurement or restricted tenure can be used as effective strategies for cost-reduction [17]. This interconnectedness shows the importance of considering access to medicines within the broader context of health systems, emphasizing the need for integrated approaches that address multiple dimensions and building blocks to ensure equitable and effective healthcare delivery. In some way, this is to be expected as the theme of interconnectivity is central to discussions in health systems research, as many frameworks developed for understanding and evaluating health systems and subsystems, such as pharmaceutical systems, integrate this theme [3, 4].

In this context, governance plays an important role in achieving universal access to medicines coordinating health system policies and defining mechanisms for ensuring availability, affordability, and appropriate use of medicines. It facilitates alignment within the pharmaceutical subsystem, which encompasses all elements involved in providing access to pharmaceutical products and services [4]. Strengthening governance involves identifying factors that hinder or promote healthcare and pharmaceutical systems’ processes and addressing them to achieve final objectives, including access to medicines. This review underscores the complexity and interdependence of the factors shaping access to essential medicines, positioning governance as a central lever for systemic improvement. Robust governance mechanisms such as transparent procurement processes, accountability frameworks for regulatory oversight, and inclusive stakeholder engagement are vital for dismantling barriers and reinforcing facilitators across pharmaceutical systems. Moreover, regional cooperation initiatives, including harmonized regulatory standards and pooled procurement strategies, further strengthen equitable and efficient healthcare delivery by fostering shared responsibility and cross-border collaboration.

### Limitations

It is important to acknowledge the weaknesses and limitations of this review. Firstly, while systematic reviews of barriers and facilitators are recurrent in the literature, they are not without known limitations, including a lack of definition, reliance on aggregative synthesis approaches, and potential oversimplification of complex social phenomena [67]. Critics argue that such reviews may overlook the interdependence of factors within complex systems and fail to consider unintended consequences [67, 68]. However, proponents highlight their utility in informing decision-making and facilitating knowledge exchange among stakeholders [67, 69]. One possible situation is that while alignment of a NEML with the WHO-EML is often seen as a facilitator, the distinct requirements of each country may supersede adherence to the WHO-EML, influenced by factors such as local needs, conditions, resources, costs, and values, which can lead to the inclusion of different medicines on their respective lists [70]. Another limitation was the methodological variations among studies, coupled with the absence of quality appraisal for this review. Diverse methods are utilized when measuring each dimension which poses challenges for comparing outcomes. Finally, despite efforts to broaden the search scope, language restrictions limited the review to English and Spanish, potentially excluding relevant studies. This may have overlooked studies in Portuguese originating from Brazil, a significant contributor to the literature on this topic.

### Conclusion

Factors such as NEML development, policies favoring generic drug procurement, and disparities between public and private sectors illustrate the complexity underlying access to essential medicines. This review underscores the critical role of strong pharmaceutical policies and regulatory frameworks in ensuring equitable access through improved availability, affordability, and appropriate use of medicines. Findings highlight the systemic interdependence among key stakeholders—governments, private sector actors, NGOs, healthcare providers, and patients—within the pharmaceutical subsystem.

To advance access across availability, affordability, and adequate use, we recommend the following actions:

- Governments should prioritize the development and enforcement of coherent pharmaceutical policies and invest in regulatory capacity-building.
- The private sector should align with national standards and promote transparency, quality assurance, and reasonable pricing across supply chains.
- NGOs can support community engagement, advocacy, and capacity-building to bridge policy and practice.
- Healthcare providers should promote rational prescribing and pharmacovigilance while serving as key links between policy and patient experience.
- Patients should be empowered through education and participation in accountability mechanisms.

Future research should explore governance innovations that improve coordination among these actors through integrated, context-sensitive approaches.
