# Supplementary material for: Access to Essential Medicines in Low- and Middle-Income Countries: A Systematic Review of Barriers and Facilitators
Source: Int J Public Health. 2026 Jan 23;71:1608754. doi: 10.3389/ijph.2026.1608754 (PMC12877563; doi:10.3389/ijph.2026.1608754)
Supplement: Supplementary file 1 [file Supplementaryfile1.docx]

Table 1 Full verbatim search strategy for PubMed, SciELO, LILACS, and Web of Science databases (Review, low- and middle- income countries, 2002-2025)

| **PubMed** | |
| --- | --- |
| ("health services accessibility"[MeSH Terms] OR "access*"[All Fields] OR "availab*"[All Fields] OR "affordab*"[All Fields] OR "rational*"[All Fields] OR "adequat*"[All Fields] OR "barrier*"[All Fields] OR "obstacle*"[All Fields] OR "restrict*"[All Fields] OR "facilitat*"[All Fields] OR "enable*"[All Fields]) AND "drugs, essential"[MeSH Terms] AND ("developing countries"[MeSH Terms] OR "LMIC"[All Fields]) | |
| **SciELO** | |
| Spanish  (Medicamentos esenciales) AND ((Accesibilidad a los Servicios de Salud) OR (acces*)) | English  (Drugs, Essential) AND ((Health Services Accessibility) OR (acces*)) |
| **LILACS** | |
| (Medicamentos Esenciales) AND ((Accesibilidad a los Servicios de Salud) OR (acceso)) | |
| **Web of Science** | |
| 1. ALL=(Drugs, Essential) 2. (ALL=(Developing Countries)) OR ALL=(LMIC) 3. (ALL=(Health Services Accessibility)) OR ALL=(acces)* 4. #3 AND #2 AND #1 | |
